# Evolutionary Instability of Symbiotic Function in *Bradyrhizobium japonicum*

**DOI:** 10.1371/journal.pone.0026370

**Published:** 2011-11-02

**Authors:** Joel L. Sachs, James E. Russell, Amanda C. Hollowell

**Affiliations:** 1 Department of Biology, University of California Riverside, Riverside, California, United States of America; 2 Institute for Integrative Genomic Sciences, University of California Riverside, Riverside, California, United States of America; Radboud University Nijmegen Medical Centre, NCMLS, The Netherlands

## Abstract

Bacterial mutualists are often acquired from the environment by eukaryotic hosts. However, both theory and empirical work suggest that this bacterial lifestyle is evolutionarily unstable. Bacterial evolution outside of the host is predicted to favor traits that promote an independent lifestyle in the environment at a cost to symbiotic function. Consistent with these predictions, environmentally-acquired bacterial mutualists often lose symbiotic function over evolutionary time. Here, we investigate the evolutionary erosion of symbiotic traits in *Bradyrhizobium japonicum*, a nodulating root symbiont of legumes. Building on a previous published phylogeny we infer loss events of nodulation capability in a natural population of *Bradyrhizobium*, potentially driven by mutation or deletion of symbiosis loci. Subsequently, we experimentally evolved representative strains from the symbiont population under host-free *in vitro* conditions to examine potential drivers of these loss events. Among *Bradyrhizobium* genotypes that evolved significant increases in fitness *in vitro*, two exhibited reduced symbiotic quality, but no experimentally evolved strain lost nodulation capability or evolved any fixed changes at six sequenced loci. Our results are consistent with trade-offs between symbiotic quality and fitness in a host free environment. However, the drivers of loss-of-nodulation events in natural *Bradyrhizobium* populations remain unknown.

## Introduction

Bacterial mutualists are often defined by their ability to form intimate and persistent infections on eukaryote hosts and to enhance host fitness [1], [2]. In most cases hosts acquire their bacterial mutualists environmentally and thus the bacteria experience selective pressure in the environment and also within the host during infection [2], [3]. Humans and intestinal flora offer an excellent example of an environmentally acquired mutualism; infants are born with a sterile gastrointestinal system that becomes infected by diverse bacterial mutualists over the first days of life [4]. Many other eukaryotic hosts acquire bacterial mutualists from environmental sources, for example legumes [5], actinorhizal plants [6], leaf-cutting ants [7], [8], tubeworms [9], stinkbugs [10], [11], bobtail squids [12] and lichenizing fungi [13] to name a few. Yet such environmentally acquired mutualisms are predicted to be evolutionarily unstable [14]–[19]. Specifically, when bacterial partners experience selective pressures during environmental life stages, adaptation to the free-living environment might come at a cost to symbiotic function [5], [20].

Consistent with the prediction of evolutionary instability [14]–[19], environmentally acquired bacterial mutualists often lose symbiotic capability over evolutionary time. Here we define symbiotic capability as the ability to form intimate and persistent interactions with hosts. Phylogenetic analyses of mutualist bacteria that encompass related environmental strains often reveal that non-symbiotic strains are nested within mutualistic-symbiont lineages [8], [11], [13], [21], [22]. These phylogenetic datasets thus infer evolutionary transitions in which bacteria lose key aspects of symbiotic function, but little is known about the molecular mechanisms that modulate these evolutionary transitions. For instance, it is unclear whether loss of symbiotic traits is driven by mutation or deletion of host-specific loci or if more widespread genomic changes are implicated. Moreover, it is unknown whether these transitions are driven by selection or drift. The selection hypothesis predicts that loss of symbiotic function is driven by a selective trade-off between environmental and symbiotic phases. This scenario requires that the maintenance of symbiotic function bears costs to bacteria in the environment, potentially enhanced by negative epistasis between environmental and symbiotic functions. The alternative hypothesis is that loss of symbiotic traits is caused by drift, for instance if long-term replication of bacterial mutualists outside the host stochastically leads to the loss of host-specific functions via mutation or deletion of loci. While the selection hypothesis predicts tradeoffs between fitness in symbiotic versus environmental phases the drift hypothesis does not predict such trade-offs.

The legume rhizobium mutualism is an excellent model system of an environmentally acquired symbiosis. Rhizobial bacteria comprise several distantly related proteobacterial lineages, most notably the genera *Azorhizobium*, *Bradyrhizobium*, *Mesorhizobium*, *Rhizobium* and *Sinorhizobium* [23], that have acquired the ability to form nodules on legumes. During infection rhizobia differentiate into specialized endosymbiotic cells called bacteroids, which fix atmospheric nitrogen in exchange for photosynthates provided by the plant [24], [25]. Rhizobial transmission among legumes is infectious [24]; legume seedlings begin life symbiont-free and must acquire their rhizobia from surrounding soils. Research focusing on *Bradyrhizobium japonicum* has uncovered abundant and highly diverse rhizobial genotypes that inhabit legume root surfaces but are non-nodulating on the *Lotus* host species from which they were cultured [5]. Some of the non-nodulating genotypes result from recent evolutionary losses of symbiotic capability, whereas other non-nodulating *Bradyrhizobium* lineages appear to exhibit little or no evolutionary history of interaction with *Lotus* hosts [22]. In *Bradyrhizobium* genomes, symbiosis loci are clustered on an integrated genomic island (a symbiosis island) that can be horizontally transferred among bacteria [22], [26], [27]. PCR analysis of *Bradyrhizobium* strains suggests that multiple loci within the symbiosis island are mutated or deleted in non-nodulating lineages; hence, that rapid evolution or deletion of the symbiosis island might drive the phenotypic loss of nodulation capability [22].

Here, we use a combination of phenotypic assays, phylogenetic reconstruction, experimental evolution, symbiotic quality assays and molecular analysis to investigate the evolutionary erosion of symbiotic function in *B. japonicum* in its interaction with *Lotus* hosts. Building on a previous dataset of 62 genotyped and phenotyped rhizobial strains [5], [22] we characterized the symbiotic quality of an additional 13 strains (nodulation capability and effects on *Lotus strigosus* growth) and sequenced two additional ‘chromosomal’ loci (e.g., not located on the symbiosis island) to reconstruct a better resolved phylogeny. We mapped the trait of nodulation capability onto the tree to resolve evolutionary loss events of symbiotic function. To infer presence or absence of the symbiosis island we used a PCR assay on multiple symbiosis loci of each strain. From the phylogeny reported here, we selected ten strains that form symbiotic nodules on *L. strigosus* and four non-nodulating strains for experimental evolution. Strains were evolved under host-free (*in vitro*) culture conditions for approximately 450 generations. Subsequently, we compared both *in vitro* fitness and symbiotic quality with *L. strigosus* between ancestral and experimentally evolved strains. Experimentally evolved strains and their ancestors were sequenced at six loci (3 chromosomal loci; 3 symbiosis loci) to analyze molecular evolution at disparate sites across the genome. Our goals are to i) estimate the evolutionary frequency of loss-of-nodulation events in a wild *Bradyrhizobium* population, ii) quantify *in vitro* fitness of symbiotic and non-symbiotic *Bradyrhizobium* isolates to examine evidence for costs of symbiotic function in a non-host environment, iii) test the hypothesis that adaptive evolution of *Bradyrhizobium* outside of the host leads to the erosion and or loss of key symbiotic phenotypes and iv) examine whether the evolutionary degradation of symbiotic phenotypes is coupled with gross changes such as deterioration or loss of symbiosis specific loci.

## Materials and Methods

### Collection of wild rhizobia and selection of isolates for phenotyping

*Bradyrhizobium* sp. were isolated from nodules and root surfaces of *L. strigosus* (49 root surface samples, 14 nodule samples, respectively), *L. heermannii* (5, 1), *L. micranthus* (3, 2), and *L. wrangelianus* (1 root-surface) in Sonoma County, California [5]. From a published phylogeny of 280 isolates [5] these representative 75 isolates were selected for phenotyping of symbiotic quality on sympatric hosts. The majority of isolates chosen for phenotyping were isolated from *L. strigosus* hosts, the host species used to assay symbiotic quality. 62 of the strains were previously phenotyped [22] and 13 additional isolates were phenotyped for this study (#'s 63–75; Table 1).

**Table 1:** Phenotypic and genotypic analysis of 75 *Bradyrhizobium* strains.

Strain Code^1^	Nod?^2^	PCR Assay^3^			Strain Code^1^	Nod?^2^	PCR Assay^3^		
		*nifD*	*nifH*	*nodDA*			*nifD*	*nifH*	*nodDA*
1_05LoS24R3.29	N	0	0	0	39_05LoS16R10.36	Y	1	1	1
2_05LoS24R3.28	Y	1	0	1	40_05LoS16R3.25	N	0	0	0
3_05LoS23R5.4	Y	1	1	1	41_05LoS16R1.17	N	0	0	0
4_05LoS21R5.36	Y	1	1	1	42_05LoS16R12.38	Y	1	1	1
5_05LoS21R1.15	N	0	0	0	43_05LoS16R8.27	N	0	0	0
6_05LoW31R2.26	N	0	0	0	44_05LoS16R2.18	N	0	0	0
7_05LoS25.1	Y	1	1	1	45_05LoS16R1.16	Y	1	0	1
8_05LoS21R5.37	N	0	0	0	46_05LoS16R2.19	Y	1	0	1
9_05LoM26R1.46	N	0	0	0	47_05LoS23R3.47	N	0	0	0
10_05LoS25R2.13	Y	1	1	1	48_05LoS25R5.28	N	0	0	0
11_05LoS23.7	Y	1	1	1	49_05LoS23R7.12	Y	1	0	1
12_05LoH15.1	Y	1	1	1	50_05LoS23R5.3	Y	1	0	1
13_05LoM26.3	Y	1	1	1	51_05LoH15R8.9	Y	1	0	1
14_05LoM26.5	Y	1	1	1	52_05LoH15R2.48	N	0	0	0
15_05LoS21R6.41	N	0	0	0	53_05LoM26R4.10	N	0	0	0
16_05LoS21.3	Y	1	1	1	54_05LoM26R2.50	N	0	0	0
17_05LoS21R1.14	N	0	0	0	55_05LoH15R5.50	Y	1	0	1
18_05LoS21R6.43	Y	1	1	1	56_05LoS22.5	Y	1	0	1
19_05LoS21R3.26	N	0	0	0	57_05LoS23R3.49	N	0	0	0
20_05LoS21R3.24	N	0	0	0	58_05LoHR2.45	N	0	0	0
21_05LoS21R3.23	N	0	0	0	59_05LoH15R8.7	N	0	0	0
22_05LoS4.2	Y	1	1	1	60_05LoS23R4.50	Y	1	1	1
23_05LoS7.4	Y	1	1	1	61_05LoS23R3.45	N	0	0	0
24_05LoS14.1	Y	1	1	1	62_05LoS24R8.1	N	0	0	0
25_05LoS21.4	Y	1	1	1	63_05LoS24R1.19	N	0	0	0
26_05LoS21R5.38	N	0	0	0	64_05LoS24R2.25	N	0	0	0
27_05LoS21R2.18	N	0	0	0	65_05LoS24R2.27	N	0	0	0
28_05LoS21R6.42	N	0	0	0	66_05LoS24R3.31	N	0	0	0
29_05LoS22R1.2	N	0	0	0	67_05LoS24R3.32	N	0	0	0
30_05LoS22.10	Y	1	1	1	68_05LoS24R5.42	N	0	0	0
31_05LoS22R3.12	Y	1	1	1	69_05LoS24R5.41	N	0	0	0
32_05LoS22R5.22	Y	1	1	1	70_05LoS25.2	Y	0	0	1
33_05LoS22R8.36	Y	1	1	1	71_05LoS25.4	Y	0	0	1
34_05LoS22R7.31	Y	1	1	1	72_05LoS25R2.15	N	0	0	0
35_05LoS3.1	Y	1	1	1	73_05LoS25R3.19	N	0	0	0
36_05LoS22.1	Y	1	1	1	74_05LoS25R5.29	N	0	0	0
37_05LoS16R10.32	Y	1	1	1	75_05LoS25R5.30	N	0	0	0
38_05LoS3.3	Y	1	1	1					

1 Strain codes include year of isolation (05 = 2005), host species (LoA = *Lotus angustissimus*, LoM = *L. micranthus*, LoH = *L. heermannii*, LoS = *L. strigosus*), plant number, and nodule or root-surface number (the latter with R followed by root and isolate number).

2 Results of greenhouse nodulation assays in which each *Bradyrhizobium* isolate was tested on 5–8 inoculated seedlings to examine nodulation capability.

3 Results of PCR amplification assays in which we attempted to amplify each of three symbiosis loci (*nifD*, *nifH*, *nodDA*). Unsuccessful reactions were repeated thrice to confirm lack of amplification.

### Symbiotic quality assays

*L. strigosus* was used as a test host for phenotyping of *Bradyrhizobium* symbiotic quality because it was the source host for the majority of the strains, but also because it is common, grows rapidly and is permissive to diverse bradyrhizobia [5], [22], [28]. Sympatric *L. strigosus* fruits were collected, seeds were surface-sterilized in bleach, rinsed in sterile ddH_2_O, nick scarified and germinated in sterile ddH_2_O. Seedlings were randomized for maternal parentage and were planted into bleach-sterilized pots filled with autoclaved quartzite sand and incubated in a growth chamber (20°C, 80% relative humidity, 12:12 day/night cycle, 2× daily misting, 14 days) before being transferred to a greenhouse under ∼50% shade for hardening (14 days, 2× daily misting). Once in the greenhouse, plants were fertilized weekly with Jensens's nitrogen-free solution [29], beginning with 1 ml per seedling, increasing by 1 ml each week until reaching 5 ml per plant, which was used thereafter. Once plants were in the greenhouse we initiated *Bradyrhizobium* cultures from ∼2 µl of original frozen stock inoculated into 50 ml of liquid MAG media [5] and incubated to logarithmic phase growth (29°C, 180 rpm, 72 hours). Bacterial concentrations were estimated via optical density on a Klett-Summerson colorimeter. Grown cultures were centrifuged (4,000 rpm, 20 min.) and re-suspended in sterile ddH_2_O (10^8^ cells ml^1^). Treatment plants were inoculated with 5 ml of re-suspended cultures and control plants were inoculated with 5 ml of sterile ddH_2_O. Each *Bradyrhizobium* isolate was tested on a group of 5–8 seedlings, with a matched number of un-inoculated controls paired by size (via a leaf count). To avoid cross-contamination during inoculation, plants were grouped by rhizobial strain. After 8 weeks of post-inoculation growth, plants were removed from pots keeping the root systems intact. Nodules (if present) were counted and dissected from roots and photographed on 1 mm grid paper. After oven drying, roots and shoots were weighed to estimate biomass.

### Reconstruction of Bradyrhizobium phylogeny and PCR analysis of symbiosis loci

To reconstruct the phylogeny we sequenced the Its locus (1233 nt; [5], [22]), GlnII (560 nt) and RecA (414 nt; [30]) for a total of 2,207 nt. We reconstructed phylogenetic relationships among 74 of the 75 phenotyped *B. japonicum* strains and three outgroup taxa: *B. japonicum* USDA110 (BA000040), *Bradyrhizobium sp.* BTAi1 (CP000494) and *Bradyrhizobium sp.* ORS278 (CU234118). One phenotyped strain (#27) was in the related nodulating lineage *Methylobacterium* and was too divergent to include on the tree. PCR fragments were sequenced using an Applied Biosystems 96 capillary 3730×l DNA Analyzer (Foster City, CA) at the Institute for Integrative Genome Biology (UC Riverside). Only unambiguous sequences in which a single nucleotide peak could be resolved for each DNA base were included in the study. All isolates were successfully sequenced and verified on Gen-Bank using BLAST [31]. Sequences were aligned using Clustal-W [32] with default parameters. Gaps were treated as missing data and only unambiguously aligned nucleotide positions were used to reconstruct phylogenies. Model fitting was performed with Mr. Modeltest 2.2 [33] and best-fit nucleotide substitution models were identified using the Akaike Information Criterion (AIC [34]). Best fit models for the Its were identified in a previous analysis [5]. For both GlnII and RecA the best-fit model was GTR + I + G. Concatenated alignments of all three loci were created and partitioned by gene, using the substitution models selected by the AIC. Phylogenetic trees were reconstructed using MrBayes 3.1.2 [35] with the following settings: eight simultaneous chains, 5•10^6^ generations, a heating temperature of 0.01, a ‘burnin’ of 9001 trees and two parallel runs starting with random trees. A plot of log-likelihood scores of sampling points (sample frequency = 100) against generation number was observed in each case to ensure that stationarity had been reached during the ‘burnin’ period. Ancestral states of nodulation ability on *L. strigosus* were inferred using a sampled set of post-burnin Bayesian trees. Ancestral states were inferred with parsimony [36], Bayesian stochastic character mapping [37], [38] and maximum likelihood [39].

Previous analysis of the original 62 isolates revealed PCR amplification success of three disparately located symbiosis loci in most isolates that successfully nodulated *L. strigosus*, including NifD (812 nt; [40]), NifH (676 nt; [30]) and an intergenic region between nodulation loci (NodD-A, 880 nt; [22]). Conversely, none of these loci successfully amplified in isolates that were non-nodulating on *L. strigosus*, which suggests that multiple symbiosis genes are mutated or that the whole symbiosis island has been deleted in these strains [22]. NifD and NifH are located at nucleotides 1,907,916–1,908,731, and 1,928,627–1,929,300 respectively, on the integrated symbiosis island of the *B. japonicum* USDA110 genome [41]. NodD-A shares only a partial homologous sequence on the USDA110 genome and is located approximately at nucleotides 1,184,000–1,185,000 also within the symbiosis island. Here, we analyzed the PCR amplification success of these three loci in the remaining 13 phenotyped strains. In all PCR experiments, successfully amplified loci were sequenced for confirmation. When PCR was unsuccessful, we repeated the reaction thrice to confirm lack of amplification, only counting attempts in which positive controls (using previously sequenced strains) were successful and negative controls (water instead of template) were blank.

### Selection of focal Bradyrhizobium strains for experimental evolution

Fourteen *Bradyrhizobium* strains were chosen from the phylogenetic analysis for experimental evolution. Strains were selected to sample the major *Bradyrhizobium* clades [5], [22] and to maximize variation in symbiotic quality on *L. strigosus* [22]. Nine of the isolates nodulate *L. strigosus* and enhance host growth compared to uninfected controls (#s 4,13,14,22,23,30,31,35, 38), one strain nodulates *L. strigosus* but provides no growth benefits to *L. strigosus* (#2), and 4 strains are unable to nodulate *L. strigosus* (#s 17,40,43,48) and exhibit no evidence of symbiosis loci in the PCR assays (Table 1 [5], [22]).

### Experimental evolution protocol

Ancestral cultures were established from frozen archived isolates (−80°C, 2∶1∶1 bacterial culture: modified arabinose gluconate media (MAG): Glycerol mixture [5]). Ancestral cultures (evolutionary cycle ‘0’) were initiated by plating colonies on solid MAG media from archives, picking single colonies for each strain and growing them individually in liquid culture to logarithmic phase (25 ml MAG media, 29°C, 180 rpm, final density ∼10^8^ cells/ml). MAG media was chosen because *B. japonicum* grows rapidly in the medium. Grown cultures were centrifuged (20 minutes, 2000 rpm) and pellets were archived (50∶50 MAG∶Glycerol, −80°C). For each strain, serial passaging was initiated with a sterile loop (∼2 ul) of evolutionary cycle 0 cells which were incubated until log phase growth was attained (25 ml MAG media, 29°C, 180 rpm; ∼96 hours). Serial transfers were conducted by taking a sterile loop (∼2 ul, ∼10^5^ cells) of the log phase culture (evolutionary cycle 1) to initiate the next evolutionary cycle. Archival samples of each cycle were created by mixing 400 µl growth culture with an equal amount MAG∶Glycerol mixture and stored at −80°C. The protocol was carried out to 30 cycles.

### *In vitro* fitness and symbiotic quality assays

*In vitro* growth rate assays were used to estimate the fitness of each experimentally evolved strain in culture at evolutionary cycles 0 and 30. Isolates were grown to log phase (∼10^8^ cells/ml), inoculated into 12 replicate cultures at low density (100 mm vials, 4 ml MAG, 10^5^ cells) and incubated (29°C, 180 rpm, 96 hours) before optical density readings were taken.

Symbiotic quality assays on *L. strigosus* were initiated in January 2010 to compare nodulation ability and symbiotic effectiveness of experimentally evolved strains (cycle 30) to their ancestors (cycle 0). Symbiotic effectiveness was measured as dry shoot biomass of infected plants compared to un-inoculated controls. We followed the same inoculation protocol as above except that ten replicate plants were used for each inoculation treatment (plus 10 size matched un-inoculated controls). Greenhouse positions of the plants were determined randomly within a two-block design. All experimental and control paired plants were separated by 15 cm and mist watered to avoid cross contamination.

### Sequence analysis of ancestral and experimentally evolved strains

For the ten experimentally evolved strains with symbiotic capability we examined genetic changes that fixed over the course of the experimental evolution. Both ancestors and experimentally evolved descendents (evolutionary cycles 0, 30) were sequenced at six loci, including the three loci used to reconstruct the phylogeny (GlnII, Its, RecA; 2,207 nt total) as well as the three symbiosis loci used in the PCR assays (NifD, NifH, NodD-A; 2,368 nt total) for a total of 4,575 nt. Any sequence differences detected between ancestral and evolved strain alignments were checked with re-sequencing.

### Data analysis

Bacterial density was assessed using optical density and the following empirically determined curve: *Bradyrhizobium* population · ml^−1^ = ((4.576 · 10^6^)(OD^culture^ − OD^blank^)−(4.632 · 10^7^)) [28]. For the *in vitro* growth assays of *Bradyrhizobium* strains we calculated isolate doubling-time using a least squares fitting exponential (http://mathworld.wolfram.com/LeastSquaresFittingExponential.html; E. W. Weisstein, From MathWorld–A Wolfram Web Resource). Two-tailed ANOVAs were used to compare growth rates between ancestral and evolved *in vitro* cultures and also to compare their relative growth effects and nodule numbers on experimentally inoculated plants. Host growth effects of the *Bradyrhizobium* inoculations were analyzed using absolute shoot and root biomass as well as a relative measure of plant growth (inoculated shoot biomass – size-matched control shoot biomass) using ANOVAs as detailed in previous studies [22], [28].

## Results

### Symbiotic quality phenotyping

*Bradyrhizobium* isolates either successfully infected *L. strigosus* by forming nodules on all the inoculated plants or failed to form nodules in any of them (Table 1, [5]). All 17 *Bradyrhizobium* isolates from nodules successfully nodulated *L. strigosus* (1 isolates from *L. heermannii*, 2 *L. micranthus*, 14 *L. strigosus*). Nineteen of 57 root-surface isolates successfully nodulated *L. strigosus* (2 isolates from *L. heermannii*, 17 *L. strigosus*). 38 strains failed to nodulate *L. strigosus* (3 isolates from *L. heermannii*, 3 *L. micranthus*, 31 *L. strigosus*, 1 *L. wrangelianus*).

### Bradyrhizobium phylogeny, ancestral state reconstruction and PCR assay

**Figure 1:**
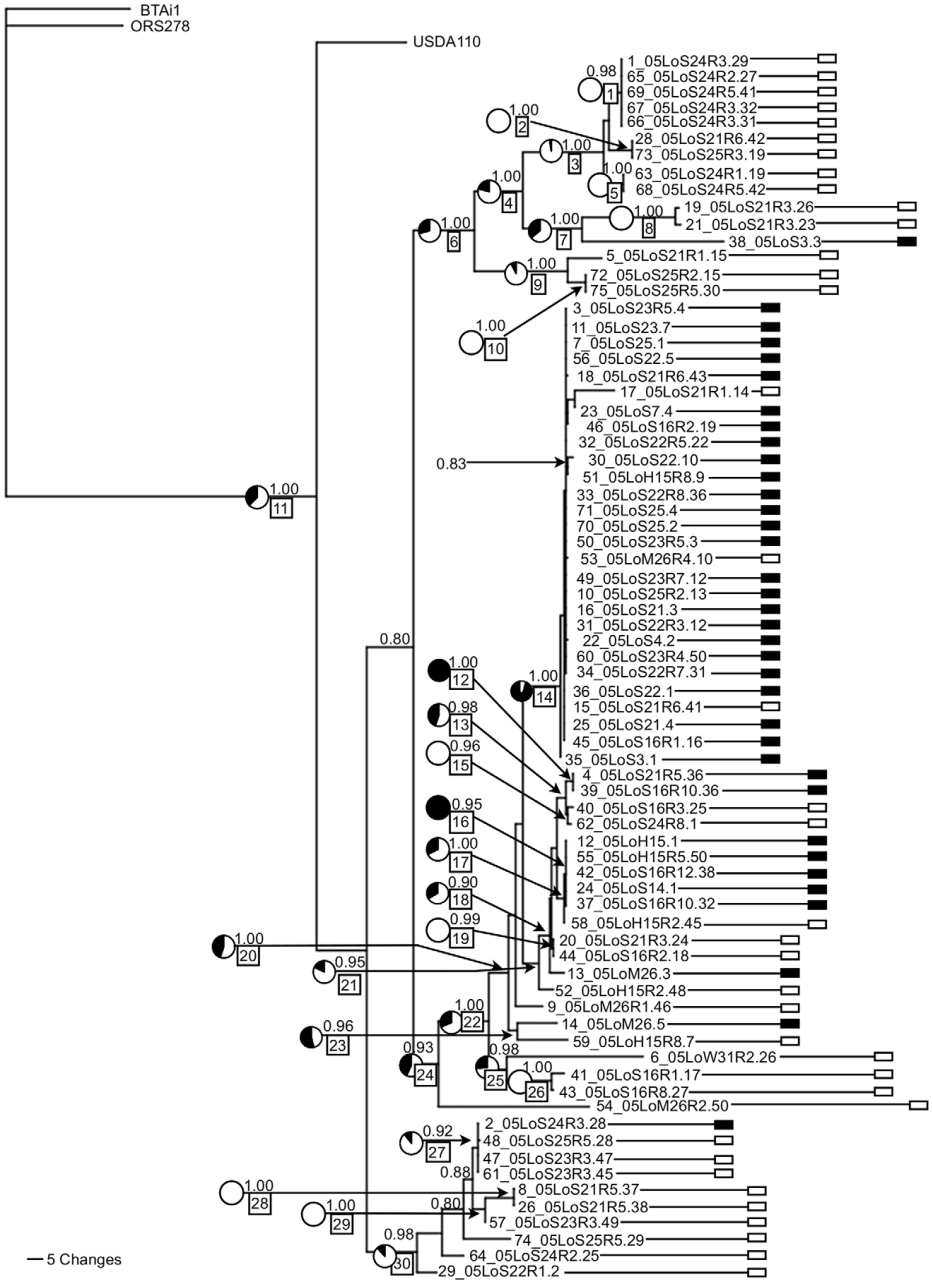
Ancestral state reconstruction of nodulation ability on *B. japonicum* phylogeny. Bayesian phylogram of 74 *Bradyrhizobium japonicum* isolates from Bodega Marine Reserve [5] inferred with three loci (Its, GlnII, RecA; total 2,238 nt) and rooted with three fully sequenced *Bradyrhizobium* strains (*B. japonicum* USDA110, *Bradyrhizobium sp.* BTAi1 and *Bradyrhizobium sp* ORS278). The tree represents a single sample from the post-burnin set of trees, in which branch lengths are scaled to indicate number of nucleotide changes. Beginning from the left, taxon labels for rhizobial isolates consist of strain number (1–75), year of isolation (05 = 2005), host species (LoA = *Lotus angustissimus*, LoM = *L. micranthus*, LoH = *L. heermannii*, LoS = *L. strigosus*), plant number, and nodule or root-surface number (the latter with R followed by root and isolate number). Strain number 27 was too divergent to include on the tree as it is more closely related to *Methylobacterium* [22]. Symbiotic phenotypes on *L. strigosus* from the inoculation assays are indicated on the tips of the tree with rectangular labels (black = nodulating on *L. strigosus*, white = non-nodulating on *L. strigosus*; [22]). Bayesian clade support values (posterior probabilities) are reported above the branches of all well-supported clades (pp≥0.80). Ancestral states are estimated for all well-supported internal nodes (pp≥0.90; labeled #s 1–30 in boxes) for the binary character of nodulating or non-nodulating on *L. strigosus* (using parsimony, maximum likelihood and Bayesian stochastic character mapping; Table 2). Bayesian posterior probabilities of the ancestral states are reported using pie charts with black filling indicating the posterior probability of the ancestor being nodulating. In the parsimony analysis all 30 well-supported ancestral nodes were inferred to be non-nodulating except for #'s 12, 14 and 16.

The *Bradyrhizobium* phylogeny is mostly well resolved, with many deep clades supported by posterior probabilities ≥0.9 (Figure 1). Only the tips of the tree tend to be unresolved, especially within the two clades that encompass many closely related symbiotic strains. *Bradyrhizobium* genotypes that nodulated or failed to nodulate *L. strigosus* are most often diverged from each other: 33 of the 36 nodulating isolates cluster into two monophyletic clades (descending from nodes #14 and #21; Figure 1) that also include ten isolates that do not nodulate *L. strigosus*. Two other well-supported clades include the majority of non-nodulating isolates (descending from nodes #6 and #30) and each of these clades also includes a single nodulating strain.

The ancestral state reconstructions (inferred using Bayesian, maximum likelihood and parsimony methods; Figure 1, Table 2) are mostly in agreement and infer that nodulation ability on *L. strigosus* has been gained and lost within the *Bradyrhizobium* population. The ancestral state reconstructions agree mostly with the previous analysis [22]. The inferred ancestral condition at the base of the tree is ambiguous, but it is more likely to be non-nodulating on *L. strigosus* based on all three analyses. At least three independent gains of nodulation capability on *L. strigosus* are apparent on the tree including lone nodulating strains nested within otherwise non-symbiotic lineages (strain #'s 2, 38 [22]) as well as ambiguous origin(s) of nodulation capability ancestral to the majority of remaining nodulating strains. Previous analyses of the three symbiosis loci (NifD, NifH, NodD-A) are consistent with gains of diverged symbiosis islands in strain #2, strain #38, and strain #'s 13 and 14 [22]. All the remaining strains exhibit symbiosis loci that are very closely related to each other [22]. We can also resolve three unique genotypes that are non-nodulating within a clade inferred to be ancestrally symbiotic (ancestral node #18; Figure 1) suggesting as many as three independent losses of nodulation capability on *L. strigosus*. The other main symbiotic clade (descending from node #21) includes a mix of nodulating and non-nodulating strains and is ambiguous in terms of multiple gains and or multiple losses of nodulation capability.

**Table 2:** Ancestral state reconstruction for supported nodes on *Bradyrhizobium* phylogeny^1^.

Node number	Clade support^2^	Pr (nodulating)^3^		Parsimony
		Bayesian	ML	
1	0.98	0.00	0.01	Non-nodulating
2	1.00	0.00	0.01	Non-nodulating
3	1.00	0.03	0.11	Non-nodulating
4	1.00	0.21	0.50	Non-nodulating
5	1.00	0.00	0.00	Non-nodulating
6	1.00	0.29	0.50	Non-nodulating
7	1.00	0.36	0.50	Non-nodulating
8	1.00	0.01	0.02	Non-nodulating
9	1.00	0.08	0.22	Non-nodulating
10	1.00	0.00	0.00	Non-nodulating
11	1.00	0.38	0.48	Non-nodulating
12	1.00	1.00	1.00	Nodulating
13	0.98	0.45	0.72	Non-nodulating
14	1.00	0.96	0.98	Nodulating
15	0.96	0.01	0.02	Non-nodulating
16	0.95	0.99	1.00	Nodulating
17	1.00	0.32	0.52	Non-nodulating
18	0.90	0.34	0.66	Non-nodulating
19	0.99	0.00	0.00	Non-nodulating
20	1.00	0.45	0.64	Non-nodulating
21	0.95	0.18	0.47	Non-nodulating
22	1.00	0.31	0.53	Non-nodulating
23	0.96	0.54	0.64	Non-nodulating
24	0.93	0.43	0.51	Non-nodulating
25	0.98	0.26	0.44	Non-nodulating
26	1.00	0.01	0.04	Non-nodulating
27	0.92	0.11	0.23	Non-nodulating
28	1.00	0.00	0.00	Non-nodulating
29	1.00	0.01	0.04	Non-nodulating
30	0.98	0.12	0.27	Non-nodulating

1 Ancestral states are inferred on the *Bradyrhizobium* phylogeny (Figure 1) for the subset of internal nodes with clade support values equal or greater than 0.9.

2 Clade support indicates the Bayesian posterior support value for each ancestral node.

3 Pr(nodulating) indicates the posterior probability of the ancestral state of nodulation estimated using both Bayesian and maximum likelihood algorithms.

We were unable to amplify any of the three symbiosis loci in any of the non-nodulating strains, consistent with previous results [22]. In the nodulating strains we were most often able to amplify all three loci and confirmed all with successful sequencing (>70% of nodulating isolates). The NodDA primers were successful with PCR and sequencing for all 36 nodulating strains, the NifD primers were successful in 34 of these strains and the NifH primers were only successful in 26 of these strains (Table 1).

### *In vitro* fitness and symbiotic quality assays

**Figure 2:**
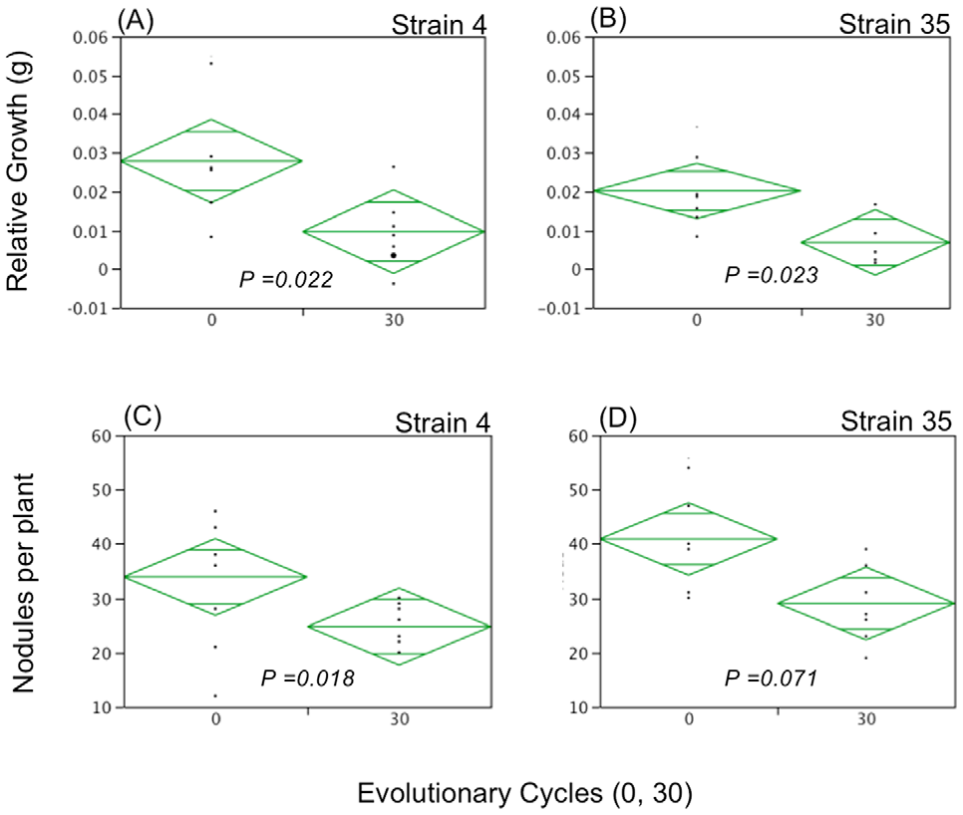
Symbiotic quality measures of ancestral and evolved *B. japonicum* isolates. Relative growth effects (panels A, B) and nodulation rates (C, D) are shown for experimentally evolved strains #4 and #35 compared to their ancestors. Relative growth effects were analyzed by subtracting the size-matched control plant shoot biomass from the shoot biomass of each inoculated plant. Nodulation rates are the number of nodules per inoculated plant. Strains #4 and #35 are shown because they exhibited the greatest evolutionary increase in *in vitro* fitness as well as the largest reduction in symbiotic quality.

Doubling time of ancestral strains ranged from ∼6.0 to 6.3 hours (Table 3). Using the maximum of 6.3 hours as a conservative doubling time estimate, we extrapolate that each culture evolved for at least 450 *in vitro* generations. Contrary to expectation, we found the ancestral mean doubling time of the ten symbiotic strains (6.128±0.011 hrs) to be significantly shorter than the mean of the isolates found to be non-symbiotic on *L. strigosus* (6.174±0.089 hrs; *F_1,158_* = 6.168; *p* = 0.014). Four of the nine analyzed strains evolved a significant increase in doubling rate over the course of the *in vitro* evolution. (#'s 4,13,22,35; Table 3). We compared symbiotic quality between ancestral and evolved isolates for these four strains. Among them, only #'s 4 and 35 evolved significant decreases in their growth benefit to hosts and only strain #35 evolved a significant decrease in the number of nodules formed on hosts (Tables 4,5; Figure 2). None of the strains evolved a significant increase in symbiotic quality during the *in vitro* evolution.

**Table 3:** Fitness evolution in *in vitro* evolved *Bradyrhizobium* isolates.

Strain Code^1^	Symbiotic Phenotype^2^	DT*^ancest^*±se^3^	DT*^evolv^*±se^3^	*F ratio* 4	*p* 4
2_05LoS24R3.28	Nodulating, Non-beneficial	6.105±0.013	6.136±0.009	F_1,23_ = 3.955	0.059
4_05LoS21R5.36	Nodulating, Beneficial	6.274±0.017	6.170±0.027	F_1,20_ = 11.15	0.004
13_05LoM26.3	Nodulating, Beneficial	6.300±0.028	6.195±0.025	F_1,19_ = 7.688	0.013
14_05LoM26.5	Nodulating, Beneficial	6.072±0.058	6.102±0.062	F_1,23_ = 1.498	0.234
22_05LoS4.2	Nodulating, Beneficial	6.063±0.021	5.983±0.020	F_1,23_ = 7.331	0.014
23_05LoS7.4	Nodulating, Beneficial	6.097±0.077	6.132±0.086	F_1,23_ = 4.459	0.291
30_05LoS22.10	Nodulating, Beneficial	6.105±0.025	6.073±0.029	F_1,22_ = 0.720	0.406
31_05LoS22R3.12	Nodulating, Beneficial	5.992±0.023	6.014±0.018	F_1,22_ = 0.556	0.464
35_05LoS3.1	Nodulating, Beneficial	6.191±0.015	6.086±0.012	F_1,21_ = 30.94	<0.001
38_05LoS3.3	Nodulating, Beneficial	6.123±0.022	NA^5^	NA^5^	NA^5^
17_05LoS21R1.14	Non-nodulating	6.223±0.018	6.186±0.027	F_1,23_ = 1.255	0
40_05LoS16R3.25	Non-nodulating	6.162±0.019	6.096±0.015	F_1,22_ = 7.538	0
43_05LoS16R8.27	Non-nodulating	6.235±0.022	6.194±0.020	F_1,22_ = 1.863	0
48_05LoS25R5.28	Non-nodulating	6.074±0.016	6.006±0.012	F_1,22_ = 11.69	0

1 Strain codes are listed as in Table 1.

2 Symbiotic phenotypes describe nodulating and growth effects status on *L. strigosus* in inoculation assays ([5], [22]).

3 Doubling (DT) time and standard error (se) are indicated for ancestral (*ancest.*) and evolved strains (*evolv.*).

4 *F* and *p* values are given for a two-tailed ANOVA comparing *in vitro* doubling time in cycle 0 and 30 cultures.

5 Evolved strain #38 is not included because it was removed due to contamination.

**Table 4:** Relative host-growth effects of ancestral versus lab evolved *Bradyrhizobium*.

Strain	Rel Grow*^ancest.^* *g* ± se^1^	*N^ancest.^* 2	Rel Grow*^evolv.^* *g* ±se^1^	*N^evolv.^* 2	*F ratio* 2	*P* 2
4	0.0278±0.0063	8	0.0096±0.0031	8	*F_1,15_ = 6.584*	0.022
13	0.0096±0.0033	8	0.0074±0.0064	7	*F_1,15_ = 0.102*	0.754
22	0.0213±0.0068	7	0.0301±0.0106	5	*F_1,11_ = 0.534*	0.480
35	0.0201±0.0037	7	0.0068±0.0028	5	*F_1,11_ = 7.173*	0.023

1 Mean relative growth effects of each *Bradyrhizobium* strain (Rel Grow) from ancestral (*ancest.*) and evolved strains (*evolv.*) is measured in grams (g) with standard error (se) by subtracting the dry biomass of control plants from their size-matched inoculated plant.

2 N, F and *p* values are given for a two-tailed ANOVA comparing relative growth effects of each strain in ancestral (*ancest.*) and evolved (*evolv.*) cultures.

**Table 5:** Biomass and nodule number of *L. strigosus* inoculated with ancestral versus *in vitro* evolved *Bradyrhizobium*.

Strain	Shoot*^ancest.^* *g* ± se^1^	*N^ancest.^* 2	Shoot*^evolv.^* *g* ± se^1^	*N^evolv.^* 2	*F ratio* 2	*P* 2
4	0.081± 0.013	8	0.048±0.007	8	*F_1,15_ = 4.845*	0.045
4c^3^	0.021±0.003	9	0.021±0.004	9	*F_1,17_ = 0.007*	0.934
13	0.046±0.011	8	0.044±0.004	9	*F_1,16_ = 0.043*	0.837
13c	0.022±0.004	10	0.012±0.003	8	*F_1,17_ = 3.899*	0.066
22	0.049±0.012	8	0.075±0.015	7	*F_1,14_ = 1.835*	0.199
22c	0.009±0.002	9	0.010±0.001	6	*F_1,14_ = 0.068*	0.799
35	0.060±0.008	8	0.036±0.005	8	*F_1,15_ = 6.635*	0.022
35c	0.006±0.001	8	0.013±0.004	5	*F_1,12_ = 4.625*	0.055

	Root*^ancest.^* *g* ± se^1^		Root*^evolv.^* *g* ± se^1^			
4	0.037±0.008	8	0.023±0.005	8	*F_1,15_ = 2.307*	0.151
4c	0.012±0.002	9	0.010±0.002	9	*F_1,17_ = 0.391*	0.541
13	0.020±0.006	8	0.020±0.003	9	*F_1,16_ = 0.001*	0.982
13c	0.010±0.003	10	0.016±0.005	8	*F_1,17_ = 1.142*	0.301
22	0.032±0.008	8	0.040±0.009	7	*F_1,14_ = 0498*	0.493
22c	0.017±0.005	9	0.016±0.002	6	*F_1,14_ = 0.012*	0.914
35	0.030±0.005	8	0.023±0.004	8	*F_1,15_ = 1.183*	0.295
35c	0.014±0.004	8	0.015±0.005	5	*F_1,12_ = 0.019*	0.893

	Nodules*^ancest.^* ± se		Nodules*^evolv.^* ± se			
4	33.88±4.44	8	24.75±1.42	8	*F_1,15_ = 3.833*	0.071
13	27.88±7.30	8	29.33±4.21	9	*F_1,16_ = 0.030*	0.866
22	26.25±5.54	8	32.14±4.01	7	*F_1,14_ = 0.705*	0.417
35	40.88±3.72	8	29.00±2.34	8	*F_1,15_ = 7.292*	0.018

1 Dry host shoot biomass (Shoot) and root biomass (Root) are measured in grams (g) with standard error (se) from infections with ancestral (*ancest.*) and evolved (*evolv.*) cultures.

2 N, F and *p* values are given for a two-tailed ANOVA comparing relative growth effects and nodules formed from infections with ancestral (*ancest.*) and evolved (*evolv.*) cultures.

3 C refers to blocks of un-inoculated control plants which showed no significant variation in growth across blocks.

### Sequence analysis of ancestral and experimentally evolved strains

Our sequence analysis of six loci revealed that no mutations fixed over the course of the experiment (in the regions that we sequenced). We did discover that evolved strain 38 was contaminated by another lineage (#35) midway through the experiment. Specifically, the sequences of evolved strain #38 were identical to the ancestral sequences of strain #35. Thus, evolved versions of strain #38 were removed from all further analyses.

## Discussion

Most beneficial bacteria are infectiously acquired [2] and thus must adapt to environments both within and outside of eukaryotic hosts. This dual lifestyle is predicted to be evolutionarily unstable because evolution that occurs outside of the host can counteract the maintenance of symbiotic function [14]–[20]. Several recent phylogenetic studies have uncovered evolutionary loss events of symbiotic function in mutualist bacterial lineages [8], [11], [13], [21], [22] that support these predictions, but the evolutionary drivers and the molecular mechanisms of these transitions remain poorly understood. In the present study we used multiple approaches to examine the evolutionary loss of legume nodulation capability in *B. japonicum*. Firstly, we bolstered data from an earlier phylogenetic analysis [22] by adding two more housekeeping loci and more bacterial isolates to the tree. Our ancestral state reconstruction is consistent with multiple evolutionary losses of nodulation capability in the sampled *Bradyrhizobium* population, although unambiguous losses can only be inferred within one clade (descendents of node #14; Figure 1). While the three non-nodulating strains that descended from this node cannot be resolved into independent monophyletic clades, they are unlikely to represent a single loss event because they are genetically distinct and at least two of them were gathered from distant sites (∼5 km [5]). The total number of loss events ranges from two to as many as six (depending on the status of ancestral node #20 for which Bayesian and maximum likelihood provide conflicting estimates of ancestral status). Our PCR analysis of three disparately located symbiosis island loci uncovered evidence for the degradation and or deletion of the symbiosis island concurrent with the phenotypic loss of nodulation ability. Specifically we were unable to PCR amplify any of the three symbiosis loci in the non-nodulating strains but we were able to amplify all three symbiosis loci in most of the nodulating strains (Table 1). The cases in which we were unable to amplify these loci in the nodulating strains is likely because of DNA variation in the PCR priming sites. Without further analysis we cannot absolutely confirm absence of symbiosis loci in any isolate, nor we cannot rule out the possibility that some symbiotic function is retained in some non-nodulating strains. Similarly, it is unclear whether degradation or wholesale loss of the symbiosis island is the driver of loss-of-nodulation status or if it occurs as an aftereffect to smaller-scale mutations that minimize or knock-out symbiotic function.

The phylogenetic pattern of symbiosis loss events that we uncovered is consistent with other research. Other bacterial mutualist lineages also exhibit symbiosis loss events that tend to be clustered near tree tips [8], [11], [13], [21]. This pattern suggests to us that the novel non-symbiotic status is itself evolutionarily unstable (either because symbiotic status is regained via horizontal gene transfer or compensatory mutation or because the non-symbiotic lineages go extinct). We uncovered rhizobial lineages that appear to be anciently non-symbiotic, similar to the findings of others [42], [43] and with few and only recent origins of nodulation capability via horizontal gene transfer [22]. Researchers have transmitted symbiosis functions to non-nodulating rhizobial strains in the lab via plasmids [42], [44], [45] and symbiosis islands [46], but our data suggest that symbiotic transfer in our *Bradyrhizobium* populations is rare in some lineages. Importantly, the non-symbiotic lineages that we identified are not lacking in access to symbiosis island DNA, since most were uncovered from the same host root surfaces as symbiotically competent strains [5], [22].

Our experimental evolution enabled investigation of the phenotypic and molecular aspects of symbiosis loss events. The phenotypic results from our *in vitro* evolved isolates support predictions of selective tradeoffs between symbiotic and environmental lifestyles [17], [18] and are not consistent with loss events being caused by drift. In particular, the two experimental *Bradyrhizobium* strains that evolved the most significant improvements in fitness *in vitro* (#'s 4,35) exhibited the greatest reduction in symbiotic quality. Yet the experimentally evolved *Bradyrhizobium* strains never lost nodulation capability as occurs in nature [22] and moreover the non-nodulating strains did not exhibit a growth advantage in *in vitro* cultures. It could be that the experimental environment did not provide the necessary selective environment to favor loss of symbiotic function. Our selection took place under nutrient rich conditions (MAG media; [5]) where bacteria were never allowed to reach stationary phase, whereas in nature *Bradyrhizobium* must survive nutrient limitation, toxicity, competition and predation between stages of host infection [20]. An alternative experiment might have evolved *B. japonicum* in media with restricted nutrients or under other sources of stress. But these stressors tend to slow growth rate and would greatly extend the time needed to complete such an experiment. A second potential reason that phenotypic evolution was relatively modest in the lab-evolved strains is that 450 generations might have been insufficient evolutionary time for loss of symbiosis to occur. The phylogenetic data suggest relatively recent loss events of both symbiosis loci and nodulation capability, but we cannot resolve the number of bacterial generations that have transpired. The degradation in symbiotic quality that we observed *in vitro* might actually precede loss of symbiotic traits in nature. For instance, some of the sister lineages to our non-fixing strains exhibited reduced symbiotic quality in greenhouse tests compared to the best symbiotic strains [22], [28].

We found no evidence of molecular evolution across six disparate loci and no loss or degradation of symbiosis loci was uncovered, inconsistent with the phylogenetic datasets. The lack of molecular evolution detected in experimentally evolved strains is likely a product of the small portion of the genome that we sequenced to analyze molecular changes (∼0.05% [41]). Previous work that has experimentally evolved bacteria and resequenced whole genomes has uncovered ∼0.002 mutations Mb^−1^ Generation^−1^ [47], [48], hence that our evolved *B. japonicum* strains might have fixed as few as ∼8 mutations per genome. In contrast, our sampling was intended to uncover gross changes such as deterioration or loss of loci. Moreover, except for the Its which encodes ribosomal DNA all of the remaining sequences mostly include coding regions, so it is possible that we missed nearby regulatory changes. It is surprising that none of the *Bradyrhizobium* strains lost symbiosis loci after 450 generations *in vitro*, especially considering the ease with which other experimenters have induced loss of rhizobial symbiotic function during *in vitro* growth [49], [50]. In these other experiments rhizobia were exposed to temperature elevation (3–6°C above our conditions) and symbiosis function was often rapidly lost via plasmid curing or deletions within symbiosis plasmids. The symbiosis island found in *Bradyrhizobium* might be more resistant to loss because it is integrated into the genome, but there is no obvious reason that it should be resistant to deletions. Evidence from the closely related symbiosis island in *Mesorhizobium* indicates that this genome region is mostly repressed in non host-associated conditions suggesting that deletions might most often be neutral *in vitro* [51].

In conclusion, we uncovered multiple evolutionary loss events of symbiotic capability in a natural population of *Bradyrhizobium*, and genotypic changes in these natural lineages indicate that symbiosis loci might be commonly lost from symbiotically effective ancestors. Our *in vitro* evolution experiment uncovered evidence of selective trade-offs between *in vitro* fitness and symbiotic quality consistent with the hypothesis that adaptation to non-host environments can drive the degradation of symbiotic quality. The experimental evolution resulted in degradation as opposed to a wholesale loss of symbiotic ability. It is possible that specific soil conditions or a longer exposure to non-host conditions would be necessary to induce loss of nodulation capability in *Bradyrhizobium*.
